# Neuropsychiatric decompensation in adolescents and adults with Phelan-McDermid syndrome: a systematic review of the literature

**DOI:** 10.1186/s13229-019-0291-3

**Published:** 2019-12-24

**Authors:** Alexander Kolevzon, Elsa Delaby, Elizabeth Berry-Kravis, Joseph D. Buxbaum, Catalina Betancur

**Affiliations:** 1 Seaver Autism Center for Research and Treatment, Icahn School of Medicine at Mount Sinai, New York, NY USA; 2 Department of Psychiatry, Icahn School of Medicine at Mount Sinai, New York, NY USA; 3 Department of Pediatrics, Icahn School of Medicine at Mount Sinai, New York, NY USA; 4 Friedman Brain Institute, Icahn School of Medicine at Mount Sinai, New York, NY USA; 5 Mindich Child Health and Development Institute, Icahn School of Medicine at Mount Sinai, New York, NY USA; 6 Sorbonne Université, INSERM, CNRS, Neuroscience Paris Seine, Institut de Biologie Paris Seine, Paris, France; 7 Department of Pediatrics, Neurological Sciences, Biochemistry, Rush University Medical Center, Chicago, Illinois USA; 8 Department of Genetics and Genomic Sciences, Icahn School of Medicine at Mount Sinai, New York, NY USA; 9 Department of Neuroscience, Icahn School of Medicine at Mount Sinai, New York, NY USA

**Keywords:** Phelan-McDermid syndrome, *SHANK3*, 22q13 deletion syndrome, Regression, Bipolar disorder, Catatonia, Psychosis

## Abstract

Phelan-McDermid syndrome (PMS) is caused by haploinsufficiency of the *SHANK3* gene on chromosome 22q13.33 and is characterized by intellectual disability, hypotonia, severe speech impairments, and autism spectrum disorder. Emerging evidence indicates that there are changes over time in the phenotype observed in individuals with PMS, including severe neuropsychiatric symptoms and loss of skills occurring in adolescence and adulthood. To gain further insight into these phenomena and to better understand the long-term course of the disorder, we conducted a systematic literature review and identified 56 PMS cases showing signs of behavioral and neurologic decompensation in adolescence or adulthood (30 females, 25 males, 1 gender unknown). Clinical presentations included features of bipolar disorder, catatonia, psychosis, and loss of skills, occurring at a mean age of 20 years. There were no apparent sex differences in the rates of these disorders except for catatonia, which appeared to be more frequent in females (13 females, 3 males). Reports of individuals with point mutations in *SHANK3* exhibiting neuropsychiatric decompensation and loss of skills demonstrate that loss of one copy of *SHANK3* is sufficient to cause these manifestations. In the majority of cases, no apparent cause could be identified; in others, symptoms appeared after acute events, such as infections, prolonged or particularly intense seizures, or changes in the individual’s environment. Several individuals had a progressive neurological deterioration, including one with juvenile onset metachromatic leukodystrophy, a severe demyelinating disorder caused by recessive mutations in the *ARSA* gene in 22q13.33. These reports provide insights into treatment options that have proven helpful in some cases, and are reviewed herein. Our survey highlights how little is currently known about neuropsychiatric presentations and loss of skills in PMS and underscores the importance of studying the natural history in individuals with PMS, including both cross-sectional and long-term longitudinal analyses. Clearer delineation of these neuropsychiatric symptoms will contribute to their recognition and prompt management and will also help uncover the underlying biological mechanisms, potentially leading to improved interventions.

## Background

Phelan-McDermid syndrome (PMS, MIM 606232) is a genetic disorder characterized by hypotonia, intellectual disability (ID), severe speech impairments, and autism spectrum disorder (ASD) [1]. Other frequently associated features include seizures, motor deficits, structural brain abnormalities, renal malformations, gastrointestinal problems, and non-specific dysmorphic features. The core neurodevelopmental features of PMS are caused by haploinsufficiency of the *SHANK3* gene, resulting from either 22q13.33 deletions encompassing *SHANK3* or point mutations of *SHANK3* [2–4]. Deletions can be either simple or result from complex rearrangements such as unbalanced translocations or ring chromosome 22.

Although the prevalence of PMS is unknown, chromosome microarray and targeted resequencing of *SHANK3* in ASD and ID suggest that up to 0.5–1% of subjects may show haploinsufficiency at this locus [5–8]. Because of its nonspecific clinical findings, the frequency of PMS is likely underestimated and is expected to increase with the widespread use of higher resolution microarrays and exome and genome sequencing with optimized coverage of *SHANK3* [6, 7]. *SHANK3* encodes a scaffolding protein that functions at excitatory postsynaptic densities to organize signaling pathways as well as the synaptic cytoskeleton [9]. In this way, the SHANK3 protein plays a critical role in glutamate transmission, synaptic spine dynamics, and, hence, in learning and memory processes.

Although the core neurobehavioral phenotype observed in individuals with PMS, including ID and ASD, has been extensively described (often in children), changes of the phenotype over time have not been well documented. In fact, little is known about the evolution of the neurological and behavioral phenotype across the lifespan, especially from a longitudinal perspective. In order to provide optimal management and follow-up of PMS patients, it will be critical to obtain insights into the natural history of PMS.

In the past few years, an increasing number of case reports described subjects with PMS showing severe regression with cognitive and/or neurological deterioration, bipolar disorder, catatonia, or psychosis arising in adolescence or adulthood [3, 10–12]. Interestingly, similar findings had been described in earlier studies, including in the first two siblings identified with a *SHANK3* mutation [2], in a patient with the smallest *SHANK3* deletion reported at the time [13], and, more than three decades ago, in individuals with ring chromosome 22 [14–16]. These descriptions converge towards a sudden change in the psychopathological presentation of the patients. The PMS family and advocacy community is also reporting such changes in social media and at family conferences, generating a great deal of concern among caregivers. It should be noted that loss of skills has also been reported to occur in early childhood in some individuals with PMS, particularly in the domains of language and previously acquired motor skills [4, 17–20]. The relationship between this early regression and later-onset phenomena is currently unknown. To gain further insight into the later-onset neurobehavioral phenotype of PMS, we conducted an exhaustive, systematic literature review of reports on individuals with PMS with signs of psychiatric decompensation, loss of skills, or sudden behavioral changes occurring in adolescence or adulthood.

## Methods

A systematic literature search was conducted looking for articles, including case reports, describing subjects with PMS showing signs of behavioral or neurologic decompensation, loss of skills, or neuropsychiatric disorders starting in adolescence or adulthood. We made use of both PubMed and Google Scholar, as well as follow-up of references cited in the papers thus identified. All relevant articles published through July 31, 2019, were included. We used different combinations of the terms Phelan-McDermid, 22q13 deletion, *SHANK3*, or ring chromosome 22, together with loss of skills/interest/abilities, regression, decline, deterioration, decompensation, catatonia, bipolar, unipolar, depression, mood swings, cyclical, hyperactivity, insomnia, manic, aggressive/aggression, outburst, tantrum, anxiety, withdrawal, apathy, agitation, oscillation, incontinence, dementia, psychosis, hallucination, and adolescent/adolescence or adult. We excluded reviews and case series that did not provide data on individual patients. To distinguish from early childhood regression, we focused on cases where the change in phenotype occurred in adolescence or adulthood.

## Results

Fifty-six cases were identified using our literature search strategy; the findings are shown in Table 1. There were 30 females and 25 males (1 unknown gender), with a mean age of 29.8 years at the time of the report (SD 12.6; range 12 to 70 years). Four families had two or three affected siblings, including three families with parental germline mosaicism and one with monozygotic twins. Earlier papers focus on subjects with ring chromosome 22, diagnosed with karyotype, before the introduction of fluorescent in situ hybridization (FISH) and later chromosomal microarrays allowed the diagnosis of terminal deletions. Ring chromosome 22 involves loss of the distal part of the long arm of the chromosome, generally involving *SHANK3* [3, 21]. More recent papers include individuals with deletions diagnosed with chromosome microarray as well as subjects with *SHANK3* point mutations. In total, there were 42 individuals with deletions (23 simple deletions, 15 ring chromosome 22, 4 unbalanced translocations), and 14 with pathogenic or likely pathogenic sequence variants in *SHANK3* (9 frameshift, 4 nonsense, and 1 missense variant).

**Table 1:** PMS patients with neuropsychiatric decompensation reported in the literature

Case	1	2	3	4	5	6
Reference	Stewart and Richards (1976) [14]^a^	Reeve et al. (1985) [15]	Arinami et al. (1986) [16] ^b^	Millichap (1994) [22]	Sovner et al. (1996) [23]	Kehrer-Sawatzki et al. (1997) [24]
Subject	−	−	−	−	−	−
Age when reported	22 y	28 y	27 y	24 y	21 y	38 y
Sex	Female	Male	Male	Female	Male	Female
Genetic abnormality	r(22), *de novo*	r(22)	r(22)	r(22)	r(22)	r(22)
Cognitive deficit, language, and behavioral problems	Severe ID, nonverbal, restless	ID, language limited to a few words. He lived at home, was described as pleasant and interacted well with his family. He worked in a sheltered workshop.	Profound ID, admitted to an institution at age 20 y	Profound ID, nonverbal, hyperactive, polyembolokoilomania	Severe ID	Mental development during early infancy was unremarkable, but language delay became apparent at the age of 4 y. She achieved low grades in primary school and was transferred to a special-needs school at the age of 10 y.
Age of onset of decompensation	18 y	~24 y	25 y	16 y	17 y	15 y
Signs of decompensation, course of illness, and treatment	At the age of 16 y, the patient had constant tremors of the head, shoulders, forearms and hands, resulting in poor motor coordination. By age 18 y, she became increasingly difficult to feed, behaviorally dysregulated, and would scream periodically. Weight loss, mental, and physical deterioration led to the need for permanent care at the age of 22 y. There remains posturing of her left forearm in a permanently flexed position with ventriflexion of the wrist. She appeared to have contractures but no neurological signs were found.	Beginning in his early 20s, the patient exhibited a deterioration of mood and behavior, with decreased speech and decline of fine motor skills over a 3 y period. He began having recurrent temper tantrums, including aggression. At other times he would withdraw, appear depressed, and become mute. He presented with truncal instability, bradykinesia, mild spastic paraparesis, resting tremor in the upper extremities, and decreased fine motor skills such that he could no longer fold papers in the vocational rehabilitation program. At the age of 27 y, agitated behavioral outbursts increased in frequency to twice daily and depression with periodic akinetic mutism continued until he required institutionalization. During hospitalization, his behavior remained unmanageable; for 1-2 h periods, he alternated between being entirely passive with avoidance of eye contact and being severely agitated, with stereotyped movements of arms and legs causing severe abrasions. All activities produced stress and increased his aggressive outbursts. He had a short attention span and could not feed himself. Psychopharmacological treatment began with thioridazine at 27 y of age with minimal benefit. He developed extrapyramidal side effects from thioridzaine within 4 weeks of increasing the dose to 1000 mg/d and abrupt discontinuation led to 4 generalized seizures. Phenobarbital, carbamazepine, chlorpromazine hydrochloride, and perphenazine were tried successively or in combination without benefit. Haloperidol worsened his agitation. A trial of methylphenidate hydrochloride resulted in rapid and dramatic improvement of his behavior within 18 h. There was further improvement as the dose was increased (eventually to 30 mg/d) along with diazepam (15 mg/d). His attention span improved so that he was able to dress, eat, and carry out personal hygiene. His mood was improved, he smiled spontaneously, and he recognized and called his family members by name. Motor function also improved, but to a lesser extent. When a dose of methylphenidate was inadvertently missed, his behavior again deteriorated, with passive withdrawal alternating with agitated, aggressive outbursts. He was eventually discharged to a residential program and while he continued to experience behavioral dysregulation and mood cycling, the frequency and severity was significantly decreased. With methylphenidate hydrochloride (40 mg/d) he had no seizures and few outbursts and was able to interact socially.	At the age of 25 y, the patient exhibited episodes of apathy, insomnia, anorexia, vomiting, mask-like facial expression, tremor of the upper extremities, instability of gait, and convulsions. These symptoms worsened and the patient died at 27 y of age. Necropsy revealed the presence of multifocal meningiomas. The apparent cause of death was a subarachnoid cyst with meningocytic elements at the medullocerebellar angle that had compressed the medulla oblongata and surrounding brain tissues. These findings in a subject with r(22) suggest a diagnosis of neurofibromatosis 2 (NF2). At the age of 20, he had no signs of NF2 on physical exam, although hearing loss was reported.	At 16 y of age, the patient was placed in a residential home because of aggressive and destructive behavior, insomnia, and refusal to eat. Self-injury was also prominent, with body bruising and insertion of foreign objects in her nose. Occasional seizures were also noted and described as “minor.” She often had a blank stare, and sometimes appeared catatonic. Behavioral modification and use of signs for communication led to improvement.	At age 17, the patient began to experience cyclical behavioral dysregulation with refusal to eat and get out of bed, sleep maintenance disturbance, decreased speech, and tearfulness. He was stabilized after approximately 2 years with what was described as a “dramatic response” to fluoxetine (40 mg/d), which led to remission of symptoms. Thirteen months later, a slow taper of fluoxetine over 3 months led to a recurrence of symptoms with anorexia, anxiety, negativistic behaviors, and self-injury (hand biting). Fluoxetine (20 mg/d) was restarted with a good initial response, but after 2 months he started to exhibit rapid cycling (3 ‘bad’ days followed by 2 ‘good’ ones). Divalproex sodium was then added. Fluoxetine (60 mg/d) in combination with divalproex sodium (level = 115 μg/ml) led to sustained remission of mood cycling symptoms.	From the age of 15 y, the patient exhibited progressive deterioration of cognitive function and mood accompanied by increasing dysarthria. At the age of 17 y, she presented with psychotic symptoms (paranoia; hallucinations) with frequent outbursts of aggressive behavior and required institutionalization. Since then, she exhibited parkinsonian symptoms with repeated akinesia. At 38 y, the patient was found to have several peripheral neurinomas and CT scan revealed bilateral vestibular schwannomas, multiple intracranial meningiomas. The MRI also showed an intraspinal tumor (T12) and an Arnold-Chiari type 1 malformation. She was diagnosed with neurofibromatosis type 2 (related to ring 22) and had neurosurgery at 38 y for excision of infratentorial tumors, a meningioma and a neurinoma. A 5-y CT follow-up showed only slight progression of the multiple supra- and infra-tentorial tumors. However, the frequency of seizures increased and she developed tetraparesis mainly affecting the lower extremities.
Proposed diagnosis on review ^c^	Unspecified catatonia	Bipolar disorder with catatonia	Bipolar disorder with catatonia, NF2	Bipolar disorder with catatonia	Bipolar disorder	Unspecified psychotic disorder, NF2
Loss of skills ^d^	+ (M, A, C)	+ (L, M, A)	+ (M)			+ (M, C)
Other information		Cranial CT scan at 27 y: diffuse mild cortical atrophy and hydrocephalus *ex vacuo*		Minor partial seizures controlled with phenytoin up to 9 y. Normal CT scan.		
Case	7	8	9	10	11	12
Reference	Anderlid et al. (2002) [13] ^e^	Ishmael et al. (2003) [25]	Ishmael et al. (2003) [25]	Ishmael et al. (2003) [25]	Tsilchorozidou et al. (2004) [26]	Nawab et al. (2007) [27]
Subject	−	Subject 1	Subject 2	Subject 5	Case 2	−
Age when reported	33 y	12 y	35 y	16 y	52 y	35 y
Sex	Female	Male	Male	Male	Female	Male
Genetic abnormality	100 kb terminal 22q13 deletion including *SHANK3*, not maternal	r(22) (p11.2;q13), *de novo*	r(22) (p11.2;q13), 80% of lymphocytes studied showed r(22)	r(22) (p12;q13)	46,XX,r(22).ish r(22)(p11q13)(TUPLE1+,10H11+,ARSA-)(47)/45,XX,-22(3)	r(22)
Cognitive deficit, language, and behavioral problems	Mild ID, speech delay, concentration difficulties at school required remedial education	ID, developmental regression after a febrile seizure at 18 m; speech regression (2-word sentences at 2 y to a few words at 5 y)	ID, nonverbal	ID, nonverbal	Severe ID, nonverbal	Severe ID, nonverbal. Long term residential care since the age of 10 y.
Age of onset of deterioration	~25 y	12 y	Teenage years	12 y	51 y	23 y
Signs of decompensation, course of illness, and treatment	At 14 y of age the patient was hospitalized due to an acute confusional state and diagnosed with epilepsy. During medication titration she had aggressive outbursts and her personality changed. In her twenties, the symptom severity progressed, and she exhibited loss of skills. By age 30, she showed features of autism spectrum disorder with lack of eye contact, stereotypic movements, and reduced expressive language. Her ability to perform daily living activities deteriorated and she also had balance problems, ataxic gait and urinary incontinence.	At 12 y of age, the patient showed significant behavioral changes and no meaningful speech. He became destructive at home, breaking objects in the house during outbursts. Psychiatric evaluation was performed and he was diagnosed with bipolar disorder and treated.	The patient had a history of behavioral problems beginning in early childhood that led to increased aggression and hyperactivity as he became older.	At 12 y of age, the patient had an episode of status epilepticus followed by chronic seizures requiring antiepileptic therapy. Mental and physical decompensation ensued. He developed sensorimotor polyneuropathy demonstrated on nerve conduction and electromyographic studies. Brain MRI showed diffuse cerebral and cerebellar atrophy. Leukocyte arylsulfatase A (ARSA) levels were low, suggesting juvenile onset metachromatic leukodystrophy; however, molecular confirmation of ARSA deficiency was not done.	The patient developed generalized seizures at 51 y (3 seizures per year). She had pronounced cyclical mood swings. At age 52 y, she had posturing suggestive of catatonia. CT revealed multiple intracranial meningiomas and cerebral atrophy. She was diagnosed with neurofibromatosis 2, related to her ring 22. As expected, mutation analysis of the *NF2* gene in blood DNA was negative. There were no cutaneous signs of neurofibromatosis and no indication for surgical treatment. She developed status epilepticus and probable aspiration pneumonia, and died shortly after the diagnosis was established.	The patient displayed cyclical changes in behavior with alternating mood shifts between mania and depression. Cycle duration varied and could occur in mixed state or change on a daily basis. During the manic phases he made noises, screamed and laughed inappropriately, was hyperactive, showed poor concentration especially while eating, and was unable to feed himself. He also had reduced need for sleep. During the depressed phases, he was lethargic, didn't eat properly, and exhibited motor retardation, apathy, and social avoidance. The intensity of symptoms worsened between age 23 y and age 35 y and his physical abilities also deteriorated. At 35 y, he was wheelchair bound. At this time, he was diagnosed with rapid cycling bipolar disorder. He had been initially treated with haloperidol with no benefit. Carbamazepine resulted in some improvement characterized by longer periods of cycling. This improvement was augmented by quetiapine but the effects were brief. Olanzapine was then used as an alternative but without benefit. Divalproex sodium eventually resulted in marked improvement.
Proposed diagnosis on review ^c^	Unspecified catatonia	Bipolar disorder	Unspecified mood disorder	Metachromatic leukodystrophy	Bipolar disorder with catatonia, NF2	Bipolar disorder with unspecified decompensation
Loss of skills ^d^	+ (L, M, A)			+ (C)		+ (M, A)
Other information	Brain CT scan at 14 y and MRI at 30 y both normal	Brain MRI at 18 months: prominent cisterna magna and mild cerebellar “atrophy”; at 12 y: giant cisterna magna and mild cerebellar “atrophy” (likely congenital cerebellar hypoplasia)				
Case	13	14	15	16	17	18
Reference	Durand et al. (2007) [2]	Durand et al. (2007) [2]	Gauthier et al. (2010) [28]	Gauthier et al. (2010) [28]	Gauthier et al. (2010) [28]	Gauthier et al. (2010) [28]
Subject	Family ASD 2 (eldest brother)	Family ASD 2 (youngest brother)	Family PED 419, subject II-1 (eldest brother)	Family PED 419, subject II-2 (middle brother)	Family PED 419, subject II-3 (youngest brother)	S00161
Age when reported	20 y	20 y	NA	NA	NA	23 y
Sex	Male	Male	Male	Male	Male	Female
Genetic abnormality	*SHANK3* frameshift mutation (NM_033517.1:c.3679dupG, p.Ala1227Glyfs*69), *de novo* (germline mosaicism)	*SHANK3* frameshift mutation (NM_033517.1:c.3679dupG, p.Ala1227Glyfs*69), *de novo* (germline mosaicism)	*SHANK3* nonsense mutation (NM_033517.1:c.3349C>T, p.Arg1117*), *de novo* (germline mosaicism)	*SHANK3* nonsense mutation (NM_033517.1:c.3349C>T, p.Arg1117*), *de novo* (germline mosaicism)	*SHANK3* nonsense mutation (NM_033517.1:c.3349C>T, p.Arg1117*), *de novo* (germline mosaicism)	*SHANK3* missense mutation (NM_033517.1:c.1606C>T, p.Arg536Trp) R536W, *de novo*
Cognitive deficit, language, and behavioral problems	ID, autism, language limited to some words and short sentences	Severe ID, autism, nonverbal	Borderline ID (IQ 72); graduated from high school in a special educational program for children with intellectual difficulties. No autistic features.	Mild ID, hyperactivity in childhood	Moderate ID (IQ 36); attended education institutions for children with intellectual deficits	Borderline ID (IQ 73), speech impairment, poor academic and social performance. No ASD traits.
Age of onset of deterioration	20 y	16 y	19 y	21 y	16 y	11 y
Signs of decompensation, course of illness, and treatment	After moving to a new residential program at 20 y of age, aggressive outbursts began with significant loss of skills, including language and toileting. He also developed anorexia and marked weight loss. At 23 y, the patient had a seizure-induced aspiration, was hospitalized, and died within a few days.	At 16 y of age, the patient had an episode of aspiration with loss of consciousness, which necessitated hospitalization. After moving to a new residential program at 16 y, loss of skills was noted in autonomy and toileting. He also developed weight loss. At 17 y of age, he developed epilepsy and was started on clonazepam. A second episode of aspiration occurred at the age of 20 y. Since then, he required a special diet with soft foods and no liquids. The patient died a few years later, after further behavioral decompensation.	Diagnosed with schizoaffective disorder	Diagnosed with schizophrenia	Diagnosed with schizophrenia	Diagnosed with schizoaffective disorder
Proposed diagnosis on review ^c^	Unspecified decompensation	Unspecified decompensation	Schizoaffective disorder	Schizophrenia	Schizophrenia	Schizoaffective disorder
Loss of skills ^d^	+ (L, A)	+ (A)				
Other information				One seizure at age 10 y.		
Case	19	20	21	22	23	24
Reference	Pasini et al. (2010) [29]	Bonaglia et al. (2011) [3]	Bonaglia et al. (2011) [3]	Willemsen et al. (2011) [30]	Verhoeven et al. (2012) [31], Egger et al. (2016) [32] ^f^	Verhoeven et al. (2012) [31], Egger et al. (2016) [32] ^g^
Subject	−	Subject P10	Subject P30	Patient 7	Patient 1 (younger brother) (Verhoeven), Patient 6 (Egger)	Patient 2 (older brother) (Verhoeven), Patient 5 (Egger)
Age when reported	18 y	40 y	40 y	48 y	29 y	31 y
Sex	Female	Female	Female	Male	Male	Male
Genetic abnormality	r(22) with 1 Mb duplication of 22q13.33 and 600 kb terminal 22q13.33 deletion, *de novo*	8.1 Mb terminal 22q13 deletion, *de novo*	3.4 Mb terminal 22q13 deletion	1.8 Mb terminal 22q13 deletion	2.15 Mb terminal 22q13 deletion, *de novo* (germline mosaicism)	2.15 Mb terminal 22q13 deletion, *de novo* (germline mosaicism)
Cognitive deficit, language, and behavioral problems	Severe ID, language limited to a few words, autistic-like behavior	Severe ID, nonverbal. Lived in an institution for the cognitively impaired	Severe ID, poor speech	Severe ID, no speech, good social interaction	Severe ID, severe speech deficit (virtually absent speech, single words only), mild features of ASD including obsessive behaviors, sleep disturbances, hyperactive behaviors with temper tantrums	Severe ID, moderately impaired development of speech and language (poor articulation, simple sentences), good social interaction, episodes of aggressive behavior
Age of onset of deterioration	17 y	39 y	39 y	45 y	17 y	27 y
Signs of decompensation, course of illness, and treatment	At the age of 17 y, the patient developed intense psychomotor agitation, severe anxiety, aggressive behavior, and insomnia. Her clinical course of illness was characterized by periods of mood cycling, hyperactivity, and self-injury. Treatment with both benzodiazepines and haloperidol was unsuccessful. Risperidone was titrated rapidly to 6 mg/d over a 2-week period and led to worsening anxiety, insomnia, and psychomotor agitation and the dose was reduced. Symptoms progressively improved at risperidone 0.5 mg twice daily. After 6 months of treatment, she showed no psychomotor agitation, aggressive behavior, anxiety, or insomnia.	The patient had her first seizure at the age of 3 y, and the second at the age of 34 y. At 39 y, her seizures became more frequent and prolonged, despite antiepileptic treatment. Neurological evaluation at age 40 y showed spastic paraparesis, with upper limbs maintained in a flexed position and flexed knees. She also showed decreased sensitivity to pain and tactile stimuli. At age 43 y, she experienced rapid motor and cognitive decline and was no longer able to stand, walk, or make eye contact. The spastic tetraparesis also markedly increased. Right renal agenesis was diagnosed during a control abdominal ultrasound and the patient died at 47 y from renal failure while in a vegetative state.	The patient had long standing epilepsy (onset at 5 y). She experienced a progressive neurological deterioration beginning at age 39 y, with cortical tremor.	The patient's general functioning began to markedly decline after a hospital admission because of severe pneumonia complicated by respiratory insufficiency at 45 y of age. He was no longer able to walk, had feeding problems due to swallowing difficulties, and became dependent on tube feeding. His social interaction also diminished. He developed seizures. Brain MRI was normal, except for mild enlargement of the cisterna magna and central atrophy. By 48 y of age, he was wheelchair dependent and showed hypertonia with spastic posture of the hands and feet. The patient died from pneumonia at the age of 49 y.	The patient presented at age 17 y with marked weight loss and behavioral changes, including loss of interest in daily activities, social withdrawal, and severe anxiety. Major depression was initially diagnosed and 2 years later, he began to show symptoms of disinhibited behavior, sleep disturbances, and compulsive rituals. Eventually irritability emerged in the context of major depression, in addition to ongoing loss of interest and marked sleep disturbance. Bipolar disorder was later diagnosed. Fluoxetine was initially started with the diagnosis of major depressive disorder but had to be stopped after 6 months because of behavioral side effects. Citalopram was later started due to worsening symptoms but it was replaced by divalproex sodium (900-1200 mg/d) in combination with haloperidol because of persistent psychomotor agitation. Despite treatment, symptoms persisted and citalopram was reintroduced without benefit. Eventually nortriptyline was added to divalproex sodium, which stabilized his mood and behavior for a period of 5 y. More recently, his treatment regimen included divalproex sodium (600 mg/d; level = 39 μg/ml) and quetiapine (800 mg/d) with stabilization of mood and behavior.	The patient first presented at the age of 27 y with an unstable pattern of mood and activity with recurrent depressive episodes. He was diagnosed with atypical bipolar disorder and treated with carbamazepine (400 mg/d) and paroxetine (30 mg/d) with good effect. This regimen was eventually replaced by divalproex sodium (900 mg/d; level = 69 μg/ml) and quetiapine (150 mg/d), which resulted in marked improvement of functioning.
Proposed diagnosis on review ^c^	Bipolar disorder	Progressive neurological disorder	Progressive neurological disorder	Progressive neurological disorder	Bipolar disorder	Bipolar disorder
Loss of skills ^d^		+ (M, C)		+ (M)		
Other information		Macrocephaly (>97^th^ centile), with normal height and weight (at age 40 y).			Brain MRI at 29 y: hypoplasia of the cerebellar vermis, enlarged cisterna magna, and mild enlargement of the lateral ventricles	Brain MRI at 31 y: hypoplasia of the cerebellar vermis, enlarged cisterna magna, and mild enlargement of the lateral ventricles
Case	25	26	27	28	29	30
Reference	Denayer et al. (2012) [11], Breckpot et al. (2016) [33]	Denayer et al. (2012) [11]	Denayer et al. (2012) [11]	Denayer et al. (2012) [11]	Vucurovic et al. (2012) [10]	Smith et al. (2012) [34]
Subject	Patient 4 (Denayer), Patient 2 (Breckpot)	Patient 5	Patient 6	Patient 7	−	−
Age when reported	25 y	43 y	46 y	51 y	18 y	23 y
Sex	Female	Female	Male	Female	Male	Female
Genetic abnormality	97 kb terminal 22q13 deletion including *SHANK3*, *de novo*	1.7 Mb terminal 22q13 deletion	1.2 Mb terminal 22q13 deletion	3.4 Mb terminal 22q13 deletion	Translocation t(14;22) with 3 small microdeletions of the *SHANK3* region and a 748 kb terminal 14q32.33 duplication. The 3 deletions occurred *de novo*.	22q13.3 deletion syndrome (no details)
Cognitive deficit, language, and behavioral problems	Severe ID, single words, ASD	Severe ID, single words	Profound ID, no speech	Profound ID, no speech	Severe ID, poor language development, inattention, hyperactive and impulsive behavior, sleep disorder associating successive hypersomnia and insomnia periods. No autistic features.	Global developmental delay, nonverbal
Age of onset of deterioration	19 y	30 y	16 y	~22 y	16 y	18 y
Signs of decompensation, course of illness, and treatment	Extreme cycling of mood and psychomotor activity, disruptive behavior, self-injury, echolalia and anxiety. The patient was diagnosed with bipolar disorder (with rapid cycling and psychotic features) at the age of 19 y. Later, symptoms of catatonia developed, in which the patient stopped moving and talking, and required tube feeding. Initial treatment with antipsychotics and benzodiazepines led to a poor response. Higher doses led to increases in body temperature and the fear of neuroleptic malignant syndrome, and neuroleptics were discontinued. Several days after discontinuation, the patient was hospitalized due to a sudden decrease in blood pressure with decreased consciousness that was thought to be due to excess benzodiazepines, but may have been a catatonic stupor, since it was followed by other signs of catatonia. Paroxetine was started. Afterwards, the patient exhibited more pronounced mood swings with fluctuating agitation, anxiety, loss of language and social skills, including no longer recognizing her mother; she stopped eating independently and lost continence. Paroxetine was stopped after her temperature rose and she became more restless. Lithium, divalproex sodium, and carbamazepine led to partial response but continued symptoms. After the diagnosis of catatonia was established, the patient was treated with lorazepam, and a few years later with ECT. Psychiatric symptoms are currently under control.	Bipolar disorder with progressive loss of skills	Mood cycling with irritability, and sleep disturbance. Bipolar disorder was diagnosed at 16 y. At 27 y of age, the patient was hospitalized in the intensive care unit due to neuroleptic malignant syndrome during a manic episode treated with high doses of haloperidol. After this hospitalization, the patient lost the ability to walk or eat independently, requiring extensive rehabilitation. At 40 y, he was hospitalized for septic shock due to aspiration pneumonia. He subsequently again lost skills of ambulation, language, eating independently, toileting, and dressing. These skills have not returned and the patient is currently spastic and wheelchair-bound despite ongoing treatment with divalproex sodium.	Diagnosed with bipolar disorder at 22 y. Experienced status epilepticus at 45 y of age and subsequently became totally dependent and bedridden.	At the age of 10 y, the patient had an episode of psychomotor agitation with insomnia lasting 3 days. At around the age of 16 y, he displayed depressive mood and social isolation. Antidepressant treatment was started and within 2 months, he became increasingly agitated with insomnia, impulsivity, and aggression. Inpatient psychiatric hospitalization was required, where he was found to be euphoric, disinhibited, with decreased need for sleep, psychomotor agitation, anorexia with weight loss, and poor attention span with concentration difficulties. His mood would also rapidly shift to depression and he was diagnosed with bipolar disorder. He developed stereotyped behavior with regression of expressive speech and lost bladder control at the age of 17. Early-onset dementia was hypothesized. Antidepressant treatment exacerbated psychomotor agitation. Carbamazepine and aripiprazole stabilized his mood changes and improved attention and concentration. Motor hyperactivity persisted.	New onset irritability, aggressive behaviors, and periodic catatonia (every 6-8 weeks) developed at the age of 18 y. Treatment with lorazepam was reportedly beneficial. ECT had been tried in the past without success.
Proposed diagnosis on review ^c^	Bipolar disorder with catatonia	Bipolar disorder	Bipolar disorder, likely brain insult secondary to septic shock	Bipolar disorder, brain insult secondary to status epilepticus or underlying cause of status epilepticus	Bipolar disorder	Unspecified catatonia
Loss of skills ^d^	+ (L, A)	+	+ (L, M, A)	+ (M, A)	+ (L, A)	
Other information	Brain MRI at 9 y and CT at 19 y were normal	Brain CT at 19, 25 and 41 y: corticosubcortical atrophy	Brain CT at 19 and 30 y: normal; at 40 y: basal ganglia infarctions	Brain CT at 43 y: mild corticosubcortical atrophy	Normal brain MRI at 16 y. Analysis of cerebrospinal fluid revealed a slight decrease in amyloid beta, low total tau and normal phosphorylated tau protein.	
Case	31	32	33	34	35	36
Reference	Verhoeven et al. (2013) [12]	Messias et al. (2013) [35], McKelvey et al. (2018) [36]	Soorya et al. (2013) [17]	Leblond et al. (2014) [7]	Guilherme et al. (2014) [37]	Serret et al. (2015) [38]
Subject	−	−	SH25	AUN_002	Patient 5	Case 1
Age when reported	70 y	41 y	45 y	NA	15 y	21 y
Sex	Female	Female	Male	Male	Male	Male
Genetic abnormality	611 kb terminal 22q13 deletion	r(22) with a terminal 22q13.32q13.33 deletion	4.4 Mb terminal 22q13 deletion, *de novo*	*SHANK3* frameshift mutation (NM_033517.1:c.4014_4015delAG, p.Gly1339Glufs*5), not maternal	46,XY,r(22)(p13q13.33) with 154 kb terminal deletion, *de novo*	*SHANK3* frameshift mutation (NM_033517.1:c.3605_3608delCCCT, p.Ser1202Cysfs*81), *de novo*
Cognitive deficit, language, and behavioral problems	Severe ID, severe language delay with monosyllabic word sentences, and behavioral problems	Moderate ID, verbal, friendly	ID, ASD, aggression	Severe ID, nonverbal, ASD	Development was unremarkable until the age of 2 y, when he presented diminished speech and social interaction. Autism	Severe ID, ASD, limited language (uses short sentences to make requests, echolalia, stereotypic language). He enjoyed sports, particularly biking.
Age of onset of deterioration	Twenties ^h^	32 y	17 y	NA	15 y	13 y
Signs of decompensation, course of illness, and treatment	Challenging, negativistic behaviors intensified over several years and necessitated institutionalization at the age of 19. During her 20s, speech became mainly incomprehensible. Mood cycling, disinhibited behaviors, and sleep disturbances markedly increased. During this period, the patient underwent several surgical corrections for painful cramping and abnormal posturing of the hands (neuroleptic-induced tardive dystonia). Over the following 3 decades, episodes of psychomotor agitation and sleep disturbances persisted. In addition, there were recurrent gastro-intestinal complaints for which no underlying cause could be found. Over subsequent years, her behavioral presentation remained mainly unchanged, although there were several periods during which inactivity was more prominent. At the age of 64 y, the patient developed mania with anxiety and agitated behavior and treatment was initiated. Despite treatment, her condition gradually deteriorated over a period of 5 y. The diagnosis of bipolar disorder was established. Treatment was started with divalproex sodium, and later replaced by carbamazepine. One year later, lithium was added. Maintenance treatment included lithium carbonate (800 mg/d; level = 0.7 mmol/l) and carbamazepine (1,000 mg/d; level = 7.1 mg/l), as well as pipamperone (40 mg tid). Due to ongoing symptoms, lithium was gradually tapered off, and staff members utilized behavioral strategies with the patient, which resulted in improvement of behavior and sleep. Carbamazepine was then reduced to 600 mg/d with pipamperone (40 mg tid) as maintenance therapy.	Mood and behavior were stable until her first psychiatric hospitalization at the age 32 y, when she began having cyclic episodes of mood dysregulation and loss of skills, including bathing, dressing, and feeding. The episodes lasted for periods of weeks, during which she became nonverbal, confused, detached, and incontinent. She often stared at her hands, at times shaking or screaming, and refused to eat. Symptoms responded poorly to SSRI or benzodiazepine monotherapy, but relatively quickly to quetiapine (300 mg twice daily), with significant improvements in affect, speech, and level of independence. Psychotic symptoms resolved and there was normalization of her sleep/wake cycle. However, cyclical episodes of depression and catatonia persisted, eventually requiring hospitalization. After admission for prolonged catatonia at age 41, a brain MRI revealed bilateral acoustic neuromas and multiple intracranial meningiomas, consistent with neurofibromatosis type 2 (related to ring 22). She had no additional physical findings of NF2. Neurosurgery was not deemed necessary. After multiple subsequent pharmacological treatments failed, she was treated with ECT with significant improvement in mood. The patient is currently stable on lithium and citalopram. She has stable mild sensorineural hearing loss, no seizure activity and no change in her tumors on annual monitoring with MRI.	Loss of language and toileting skills after being placed in a residential program.	The patient began experiencing generalized tonic-clonic seizures at 8 y, which were resistant to treatment. Regression was reported late in life (no other information available), in addition to ataxia and dysmetria.	New onset aggression after he started having seizures at the age of 15 y. Seizures were controlled with carbamazepine; aggressive behavior was treated with haloperidol and risperidone.	After moving from an autism day-care center to an autism unit at 13 y, the patient showed progressively reduced motor, verbal, and daily living skills. He stopped his favorite activities and became behaviorally dysregulated, with increased impulsivity, negativistic behavior, and apathy. Sleep disturbance with insomnia appeared. At age 15 y, the patient required hospitalization in a child and adolescent psychiatric unit. The patient received different psychotropic medications that were partially effective but accompanied by multiple side effects. Antipsychotics were able to control aggressive behaviors and insomnia, but induced catatonia and elevated muscle enzymes. Benzodiazepines reduced catatonia symptoms but didn't control impulsivity, and increased psychomotor agitation, confusion, and insomnia. Mood stabilizers had no clinical efficacy and induced side effects, such as DRESS syndrome (Drug Reaction with Eosinophilia and Systemic Symptoms) with carbamazepine. Antidepressants were not effective. Among multiple drug associations, only the combination of aripiprazole and clonazepam showed partial efficacy, facilitating the improvement in daily living, language, and motor skills for about 12 months, but with no impact on behavior. Methylphenidate was not effective and induced insomnia, violence and shouting. Lithium was initially introduced in association with other psychotropic medications (e.g., methylphenidate) and for a short period (<2 months), without clear clinical effects. At age 19, there was no significant improvement, and the diagnosis of bipolar disorder was suspected. Lithium (1500 mg/d) and melatonin (4 mg/d) led to symptom stabilization over 3 months. Agitation, aggressiveness and impulsivity disappeared. The patient regained autonomy in everyday life, and urinary and bowel incontinence stopped. He no longer showed opposition, participated in activities with other patients, and began riding his bicycle again. As he no longer had sleep disturbances, melatonin was discontinued after 3 months. One year after the beginning of lithium therapy, the patient moved to an adult group home and had recovered his baseline level of functioning. At a follow-up of 2 y, he remained stable on lithium (1500 mg/d; level = 0.8 mEq/l).
Proposed diagnosis on review ^c^	Bipolar disorder with catatonia	Bipolar disorder with catatonia, NF2	Unspecified decompensation	Likely neurological disorder	Unspecified decompensation	Bipolar disorder with catatonia
Loss of skills ^d^	+ (L)	+ (L, A)	+ (L, A)	+ (M)		+ (L, M, A)
Other information	Brain MRI at 70 y: cortical atrophy, particularly in the frontal region, and subtle periventricular white matter changes				Normal head CT	Normal brain MRI and EEG
Case	37	38	39	40	41	42
Reference	Serret et al. (2015) [38] ^i^	Egger et al. (2016) [32]	Egger et al. (2016) [32]	Egger et al. (2016) [32]	Egger et al. (2016) [32]	Egger et al. (2016) [32]
Subject	Case 2	Patient 1	Patient 2	Patient 3	Patient 4	Patient 7
Age when reported	17 y	44 y	22 y	33 y	23 y	21 y
Sex	Female	Male	Female	Female	Female	Female
Genetic abnormality	*SHANK3* nonsense mutation (NM_033517.1:c.2425G>T, p.Glu809*), *de novo*	63 kb 22q13.33 deletion including *SHANK3*	Unbalanced translocation 11;22 with derivative chromosome 22, 11q24.2q25 duplication of 8.77 Mb and terminal 22q13.33 deletion of 512 kb	1.98 Mb 22q13.32q13.33 deletion	Unbalanced translocation 8;22 with terminal 22q13.33 deletion, *de novo*	88 kb 22q13.33 deletion including *SHANK3*
Cognitive deficit, language, and behavioral problems	Severe ID, ASD. She presented echolalia and stereotypic language with words and short sentences used in everyday life and in context. She enjoyed sports, especially rock climbing and gymnastics.	Profound ID, virtually absent speech, single words only	Mild to moderate ID, limited speech and receptive language, ritualistic/compulsive behaviors, sleep disturbances. Attended special education until the age of 18 y.	Mild to moderate ID, poor articulation, simple sentences	Profound ID, absent speech	Mild to moderate ID, elementary sentences
Age of onset of deterioration	12 y	Late adolescence ^j^	Late adolescence	Late adolescence ^j^	Late adolescence ^j^	Late adolescence ^j^
Signs of decompensation, course of illness, and treatment	Major clinical changes were observed after the patient left her day-care center for an autism unit at age 12. She exhibited behavioral dysregulation, lost toileting skills, and slowly lost motor and verbal skills. She developed impulsive and aggressive behaviors. Her posture became hunched and she remained frequently motionless, needing physical stimulation in order to move. This was followed by further deterioration of skills, incontinence and insomnia. By age 16 y, the diagnosis of catatonia-like symptoms was considered and treatment with clonazepam (0.9 mg/d) was started. The patient rapidly improved and in a few months regained motor abilities, normal body posture, language, participation in activities, and autonomy. Sleep disturbances and fecal incontinence stopped but nocturnal enuresis persisted. These improvements continued for about 8 months. However, psychomotor agitation and increased verbal output progressively increased. She lost weight while continuing to eat normally (lost 8 kg in 8 months), so clonazepam was progressively tapered. During the next 4 months, she regained weight, but then regressed again by losing motor and verbal skills. She presented once again with catatonia-like symptoms including aggressiveness and insomnia. Clonazepam (0.9 mg/d) was reintroduced but led to psychomotor agitation, increased verbal output, impulsivity, and insomnia, necessitating psychiatric hospitalization. Clonazepam was switched to lorazepam (3 mg/d), complicated by confusion, fecal incontinence, agitation and insomnia. By age 17 y, the diagnosis of bipolar disorder was established and lithium (1000 mg/d; level = 0.7 mEq/l) was started with clonazepam (0.2 mg/d). The behavioral symptoms stabilized and she exhibited improved verbal and motor abilities, participation, sleep and autonomy. However, nocturnal enuresis persisted. Clonazepam was discontinued after 4 months. After 1 year of lithium treatment, the behavioral dysregulation stabilized and the patient returned to her baseline level of functioning, without adverse effects.	Diagnosed with bipolar disorder. Treatment with pipamperone (120 mg/d), lamotrigine (350 mg/d), and levothyroxine (125 μg/d) resulted in marked stabilization of mood and behavior.	Beginning in late adolescence, the patient had recurrent mood changes paralleled by an increase of pre-existing autistic behaviors. Treatment with paroxetine resulted in behavioral deterioration with possible hallucinations. On addition of haloperidol, later replaced by risperidone, the patient developed a serotonin syndrome for which she was hospitalized. Discontinuation of all psychotropic medication and symptomatic treatment resulted in a rapid remission of symptoms and she was discharged to her parent’s home. A urinary tract infection diagnosed during the hospitalization was thought to have contributed to her initial deterioration of behavior. Because her general functioning did not reach baseline levels she was referred for evaluation and diagnosed with bipolar disorder. Treatment with divalproex sodium was started. After 6 months, a dose of 600 mg/d (level = 45 μg/ml) resulted in notable stabilization of mood and behavior, although baseline levels of function were still not fully attained and the addition of quetiapine is being considered.	Diagnosed with bipolar disorder. Treatment with divalproex sodium (600 mg/d) and quetiapine (300 mg/d) resulted in marked improvement of functioning.	Diagnosed with bipolar disorder	Diagnosed with bipolar disorder. Treatment with quetiapine (1000 mg/d) resulted in marked improvement of functioning.
Proposed diagnosis on review ^c^	Bipolar disorder with catatonia	Bipolar disorder	Bipolar disorder	Bipolar disorder	Bipolar disorder	Bipolar disorder
Loss of skills ^d^	+ (L, M, A)					
Other information	Brain MRI, EEG and biological analyses performed at the time of regression were normal		Normal brain MRI at age 22 y	Brain MRI: Hypoplasia of cerebellar vermis and mild ventricular enlargement		
Case	43	44	45	46	47	48
Reference	Breckpot et al. (2016) [33]	Fokstuen et al. (2016) [39]	Egger et al. (2017) [40]	Tabet et al. (2017) [41]	Tabet et al. (2017) [41]	Tabet et al. (2017) [41]
Subject	Patient 1	−	−	P3	P9	P11
Age when reported	46 y	NA	43 y	16 y	27 y	37 y
Sex	Female	NA	Male	Female ^k^	Female	Female
Genetic abnormality	97 kb 22q13.33 deletion including *SHANK3*, *de novo*	*SHANK3* frameshift mutation (NM_033517.1:c.3637dupC, p.His1213Profs*83)	*SHANK3* frameshift mutation (NM_033517.1:c.4523delC, p.Thr1508Serfs*36)	22q13.33 deletion diagnosed by FISH, *de novo*	Unbalanced translocation t(2;22) with 5.07 Mb terminal 22q13 deletion, paternally derived	716 kb 22q13 terminal deletion, *de novo*
Cognitive deficit, language, and behavioral problems	Profound ID, autistic behavior	ID, mutism	Severe ID, limited verbal skills, ASD; destructive, self-injurious and withdrawal behaviors during childhood	ID, non verbal (language regression at 18 m), autistic traits	ID, non verbal, oppositional defiant disorder	ID, speech delay, no autistic traits
Age of onset of deterioration	20 y ^j^	NA	~16 y	22 y	17 y	NA
Signs of decompensation, course of illness, and treatment	Diagnosed with unspecified psychosis and catatonia. Inpatient in an adult psychiatric unit. The first signs of severe regression started around the age of 20 y. The first catatonic episode was treated with lorazepam, the second with ECT, with good response. Although ECT resulted in remission of the catatonic symptoms, the psychotic symptoms remain, with inconsistent response to psychopharmacological treatment.	Unspecified psychotic symptoms	After being institutionalized at age 9 y, the patient‘s general functioning remained relatively adequate over the following years. Subsequent to an institutional reorganization, his behavior decompensated with loss of language, motor functioning, and continence. At 16 y, there was severe mood and behavior dysregulation with sleep disturbance and anxiety. Over the following decade, the patient had sustained sleep disturbance and alternating episodes of apathy, impulsivity, food refusal and weight loss. Bipolar disorder (with rapid cycling features) was suspected. Several psychotropic medications were tried with equivocal results and poor tolerability. At 24 y, treatment with carbamazepine and divalproex sodium was prescribed with limited benefit. Lamotrigine and olanzapine were reported to induce tardive dyskinesia. Olanzapine was replaced by clozapine, which induced severe constipation, recurrent seizures, and agranulocytosis. Treatment was then restricted to clorazepate. At age 42 y, olanzapine, divalproex sodium, and lamotrigine were started, but the instability persisted, with intermittent aggressive and apathetic episodes. After the diagnosis of PMS due to a *SHANK3* mutation was established at age 44 y, lithium was started and titrated to 700 mg/d (level = 0.5 μg/ml) in addition to olanzapine (10 mg/d). Over the course of several months, lithium resulted in marked stabilization of mood and behavior.	Severe hyperactivity and lack of sleep with subsequent loss of skills, particularly speech at the age of 22 y.	Severe status epilepticus at age 17 y despite treatment with antiepileptic medication, leading to loss of cognitive skills, loss of visual acuity, and loss of locomotion.	Diagnosed with bipolar disorder and perceptual disturbances (hallucinations).
Proposed diagnosis on review ^c^	Unspecified psychotic disorder with catatonia	Unspecified psychotic disorder	Bipolar disorder	Unspecified decompensation	Unspecified decompensation	Bipolar disorder
Loss of skills ^d^	+		+ (L, M, A)	+ (L)	+ (M, C)	
Other information	Cerebral and cerebellar atrophy; postoperative seizure		Brain MRI at 42 y: discrete loss of cerebral tissue predominantly in the left hemisphere with enlarged sulci.			
Case	49	50	51	52	53	54
Reference	Tabet et al. (2017) [41]	Ballesteros et al. (2017) [42]	Lyons-Warren et al. (2017) [43]	De Rubeis et al. (2018) [4]	De Rubeis et al. (2018) [4]	De Rubeis et al. (2018) [4]
Subject	P83	−	−	S12	B2 (MZ twin)	B3 (MZ twin)
Age when reported	23 y	13 y	16 y	42 y	14 y	14 y
Sex	Male	Female	Female	Female	Female	Female
Genetic abnormality	619 kb 22q13 terminal deletion, *de novo*	2.7 Mb 22q13 terminal deletion ^l^	r(22) with 1.036 Mb terminal 22q13 deletion	*SHANK3* frameshift mutation (NM_033517.1: c.4906_4921dupTCCCCCTCGCCGTCGC, p.Pro1641Leufs*58), de novo	*SHANK3* frameshift mutation (NM_033517.1: c.4065_4066delTG, p.Val1357Glyfs*4), de novo	*SHANK3* frameshift mutation (NM_033517.1: c.4065_4066delTG, p.Val1357Glyfs*4), de novo
Cognitive deficit, language, and behavioral problems	ID, speech delay, ASD, ADHD, aggressive behavior	ID, ASD	ID, ASD; as a child, loss of previously mastered vocabulary	Profound ID, verbally fluent until age 12-13 y	Mild ID, speaks in full sentences but developed word finding difficulties. No ASD. Aggression.	Mild ID. Spoke in full sentences but regressed at 9 y to only say 2-3 words, regained some vocabulary but fluctuating language. No ASD. Aggression.
Age of onset of deterioration	20 y	11 y	16 y	12-13 y	13 y	9-10 y
Signs of decompensation, course of illness, and treatment	Loss of cognitive skills with worsening social communication impairment and increased stereotyped behaviors at 20 y.	Behavioral dysregulation with catatonic features and regression at age 11 y that required psychiatric hospitalization. Different pharmacological treatments, such as antipsychotics and benzodiazepines, failed to improve symptoms and led to multiple adverse events. Lithium therapy stabilized behavioral symptoms and allowed the patient to recover her baseline level of functioning. After the first menstruation there was cycling through periods; risperidone was suggested as adjunctive therapy but the patient later stabilized with lithium monotherapy.	Symptoms began at age 16 y with a personality change and subsequent episodes of unprovoked anger. The patient developed painless growths on both arms. Brain MRI revealed bilateral vestibular and spinal schwannomas (which had not been observed when MRI was performed at 8 y), consistent with a diagnosis of neurofibromatosis type 2 (related to ring 22). EEG revealed an epileptogenic focus over the left temporal region. The patient subsequently had 1 episode of suspected seizure. She was treated with bevacizumab with significant improvement in her mood.	Intermittent periods of behavioral dysregulation with loss of cognitive, motor and language skills. Sometimes preceded by viral infection. Cognitive ability declined from borderline intellectual functioning before puberty to profound ID at age 42. The patient was verbally fluent but became non-verbal. She was walking independently at 20 months but currently is unable to walk more than several steps without support. Psychotic symptoms were reported and characterized by auditory and visual hallucinations. She had episodic periods of mania and depression, insomnia, decreased appetite and weight loss, unsteady gait, and catatonic posturing.	“Manic like” behavior	“Manic like” behavior
Proposed diagnosis on review ^c^	Unspecified decompensation	Bipolar disorder with catatonia	Unspecified decompensation, NF2	Bipolar disorder with catatonia	Unspecified mood disorder	Unspecified mood disorder
Loss of skills ^d^	+ (L, C)	+		+ (L, M, C)		
Other information				Normal brain MRI at 14 y and 18 y. Macrocephaly (OFC 57 cm, 99^th^ centile), with normal height and weight (at age 42 y).	Brain MRI at 14 y: mild cerebellar tonsillar ectopia. Atypical absence seizures, onset at 14 y. Episode of idiopathic intracranial hypertension at 12 y. Normal OFC (30^th^ centile) at 14 y.	Normal brain MRI at 14 y. Atypical absence and tonic seizures, onset at 7 y. Macrocephaly (OFC 57 cm, 98^th^ centile) at 14 y.
Case	55	56				
Reference	Kildahl et al. (2018) [44]	Jungová et al. (2018) [45]				
Subject	−	−				
Age when reported	22 y	30 y				
Sex	Male	Female				
Genetic abnormality	30 kb interstitial deletion extending from *SHANK3* to *ACR*	54 kb deletion partially overlapping *SHANK3*, de novo				
Cognitive deficit, language, and behavioral problems	Severe ID, ASD, language limited to simple phrases	Mild speech delay, spoke in full sentences; no autistic traits. Because of low grades in primary school, she was transferred to a special-needs school at the age of 10 y. IQ 60 at 15 y. After finishing school she had a part-time job as a manual worker.				
Age of onset of deterioration	Late teens	23 y				
Signs of decompensation, course of illness, and treatment	Behavioral changes observed during the late teens, with a cyclical pattern of increased motor activity alternating with periods of decreased activity. At 22 y, the patient was hospitalized and received the diagnoses of ID, ASD, and bipolar disorder. Several treatments were initially attempted without success, including olanzapine, levopromazine for agitation, alimethazine for sleep, and mirtazapine for depression. At the time he received the diagnosis of bipolar disorder, divalproex sodium treatment was initiated. The patient became more calm and exhibited increased social interaction. Although episodes of psychomotor agitation continued, they were less severe and his mood was more stable. The patient also now receives more adapted care, particularly regarding his underlying ASD symptoms.	The patient was hospitalized at 23 y because of an acute psychotic episode with agitation and insomnia during a respiratory infection with fever (38.5°C). She was treated with olanzapine, benzodiazepine, and maprotiline, and discharged 3 weeks later with full recovery. From the age of 25, she had intermittent loss of bladder control, memory impairment, inattention, partial loss of independence, easy fatigability, and loss of language skills - screaming meaningless words and repeating the same sentences. These behavioral changes were triggered by mild febrile episodes (37–38°C); they occurred initially 3-4 times per year, and later around once a month. The fever was sometimes associated with mild pharyngitis but other times the cause was unclear. No depressive symptoms were observed. Maprotiline was suspended for possible side effects, olanzapine was continued with good effect but was later replaced by quetiapine due to excessive weight gain. Between the ages of 27 and 30 y, the patient was admitted to the hospital 5 times for severe episodes, diagnosed as either psychotic disorder or bipolar disorder. The episodes began with insomnia and incoherence soon after the onset of fever. Examination also revealed pychomotor agitation, instability, mutism (little to no verbal response), screaming, “delusions,” and “intermittent spastic paraparesis of the upper left extremity.” These episodes continued after the fever subsided and lasted 1-3 weeks. Treatment with divalproex sodium, chlorprothixene and benzodiazepine was effective for remission of psychosis, mood, and behavior dysregulation. After recovery, she returned to her previous level of functioning, except after the fifth hospitalization. This episode was the most severe; she was unable to understand instructions, displayed rigidity, catatonia, and had to be catheterized due to urinary retention. After discharge, her cognitive and verbal abilities worsened, and she now requires instructions from her parents to perform basic activities.				
Proposed diagnosis on review ^c^	Bipolar disorder	Bipolar disorder with catatonia				
Loss of skills ^d^		+ (L, A, C)				
Other information		Extensive medical investigations over the years were non contributory. Brain MRI showed incipient cortical atrophy.				

*ADHD* attention deficit hyperactivity disorder, *ASD* autism spectrum disorder, *CT* computed tomography, *ECT* electroconvulsive therapy, *EEG* electroencephalography, *ID* intellectual disability, *kb* kilobase, *Mb* megabase, *MZ* monozygotic, *MRI* magnetic resonance imaging, *NA* not available, *NF*2 neurofibromatosis type 2, *OFC* occipito-frontal circumference, *PMS* Phelan-McDermid syndrome, *r(22)* ring chromosome 22, *SSRI* selective serotonin reuptake inhibitor, *tid* 3 times a day, *y* years

^a^Patient initially described by Richards et al. [46]

^b^Patient initially reported by Kondo et al. [47] (Case 5)

^c^The diagnosis reported in the publication is mentioned above, in ‘Signs of decompensation, course of illness, and treatment’, when available.

^d^Loss of skills: L=language, M=motor, A=activities of daily living, C=cognitive

^e^Patient also reported very briefly in a study of subtelomeric rearrangements [48]

^f^Subject also reported by Willemsen et al. [30] (Patient 9)

^g^Subject also reported by Willemsen et al. [30] (Patient 8)

^h^Neuropsychiatric decompensation was reported to have occurred during the “second decade” in the original article; when contacted, the authors stated that it occurred during her twenties.

^i^Patient also reported by Leblond et al. [7] (AUN-003) and Darville et al. [49] (Patient 1)

^j^Information provided by author

^k^Patient listed as male in Table S2 but referred to as ‘her/she’ in the clinical description in the supplement

^l^Patient was mistakenly reported to have a mutation in *SHANK3*; she has a 22q13 terminal deletion

**Fig. 1:**
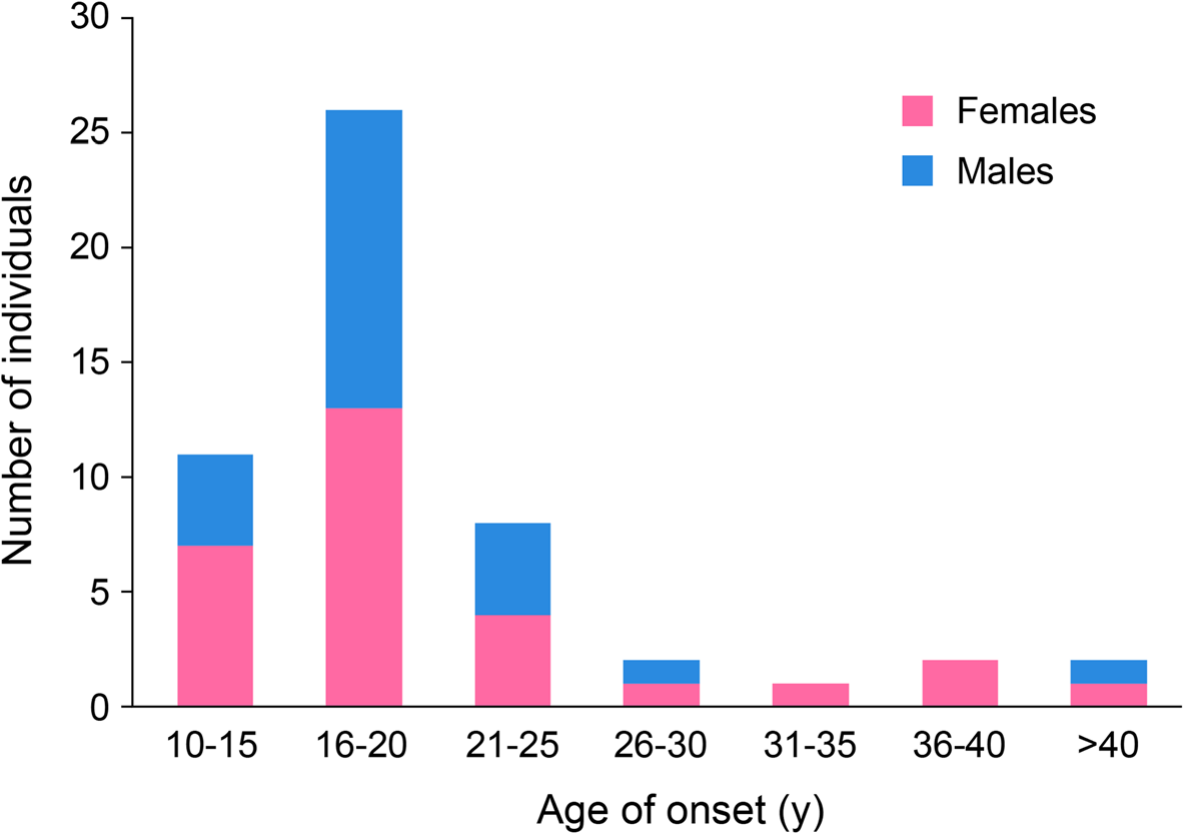
Age of onset of regression or emergent psychiatric phenotypes. For each patient report where the onset of regression or the emergence of psychiatric phenotypes was clearly documented, we noted the age and summed the number of individuals in each bin. We omitted all cases without such information. Cases with onset in “late adolescence” or “late teens” were included in the 16–20 years bin (see Table 1). For those cases with a 2-year window of onset (i.e., 9–10 and 12–13), we used the later time point. Females and males were counted together but identified by differing colors

Some reports have limited descriptions of the subjects, while others present a complete clinical evaluation. All individuals had ID, which was generally severe (20 out of 40); 8 had profound ID, 5 mild to moderate ID, 5 mild ID, and 2 had borderline IQ (no information about the level of ID was available for 16 individuals). Although language impairment was prominent, several individuals were reported to speak in full sentences at baseline. The mean age of onset of neuropsychiatric decompensation was 20 years (SD 8.4); the youngest patient showed changes at 9-10 years of age (P54) and the oldest at 51 years (P11). In 71% of the patients, the onset of neuropsychiatric symptoms occurred between the ages of 9 and 20, with a peak of onset at 16–20 years (Fig. 1). Although samples were small, there was no evidence of a sex difference in the age of onset (Fig. 1).

Thirty-one individuals exhibited significant loss of skills (17 females, 14 males) with a mean age at onset of 21 years. Thirty individuals had bipolar disorder (17 females, 13 males; mean age at onset 20 years); catatonia was reported in 16 (13 females, 3 males; mean age at onset 22 years), and psychosis in 7 (3 females, 3 males, 1 unknown gender; mean age at onset 17 years). Three patients had an unspecified mood disorder (2 females, 1 male; mean age at onset 11 years). At least four individuals had a progressive neurological disorder (2 females, 2 males), with juvenile onset in one (12 years) and adult onset in three (mean age 41 years). In addition, there were eight patients with unspecified decompensation and one with a likely neurological disorder, not included in the previous categories (3 females, 6 males; mean age at onset 18 years).

### Loss of skills

Significant loss of skills was reported in 31 of 56 (55%) individuals. Loss of skills is often referred to as “regression” in the literature reviewed but the details provided in most of the case reports do not clarify whether individuals clearly and consistently acquired skills for a prolonged period of time and then lost these skills, either permanently or for an extended period. In general, neuropsychiatric disorders such as bipolar disorder, catatonia, and psychosis may emerge with a loss of skills but most of the available reports do not clarify whether symptoms persisted beyond the acute psychiatric episodes. Loss of skills occurred in a variety of areas, most commonly affecting language (16 of 26 with information, 62%) (for specific patient and types of loss of skills see Table 1), motor skills (16 of 27, 59%), and activities of daily living, including toileting skills (16 of 26, 62%). Cognition was also reportedly affected in many cases (8 of 26, 31%). Motor skill loss was dramatic in several cases, leading individuals to be unable to walk in two cases (P20, P47), wheelchair bound in three cases (P12, P22, P27), or bedridden in one case (P28).

### Bipolar disorder

Among the cases we reviewed, 30 of 56 (54%) most likely met criteria for bipolar disorder. As with all psychiatric disorders, reliable diagnosis is challenging in intellectually disabled and minimally verbal individuals. Relying on the descriptions provided in the literature, however, several themes were common among individuals with PMS, consistent with the diagnosis of bipolar disorder. Among them, irritability, mood cycling or mood dysregulation was described in most (*n* = 20). Sleep was also highly disturbed in many (*n* = 16), with decreased need for sleep, insomnia, and sleep maintenance problems. Distractibility or short attention span was noted in at least four patients. Some patients were described as screaming (*n* = 3) or hyperactive during periods (*n* = 3). Loss of skills was also commonly associated, with 50% (15 of 30) of those with bipolar symptoms also having loss of function (Table 1), such as loss of language (*n* = 11), motor skills (*n* = 9), bathing and dressing skills (*n* = 1), weight loss/feeding issues (*n* = 9), cognition (*n* = 2), and continence (*n* = 6). Rapid cycling was noted in five individuals. Seven patients had symptoms where the severity reached the need for hospitalization. Fever or infection (P39, P52, P56) and first menses (P50) were potential antecedents.

A broad range of medications typically used for bipolar disorder were administered in most cases, but met with inconsistent success in PMS. Antipsychotics were most commonly prescribed, such as thioridazine, chlorpromazine, perphenazine, haloperidol, chlorprothixene, pipamperone, risperidone, olanzapine, aripiprazole, and quetiapine, either alone or in combination with anticonvulsants and/or benzodiazepines. No clear themes of effectiveness are evident based on our review, and if anything, antipsychotics were generally ineffective and often poorly tolerated. In one notable case (P19), different therapeutic responses were observed between low- and high-dose risperidone; high dose (6 mg daily) resulted in poor response and increased behavioral symptoms, while low dose (1 mg daily) improved mood and behavior. In several cases, the combination of an antipsychotic and anticonvulsant, such as quetiapine with divalproex sodium (P23, P24, P40, P42), aripiprazole and carbamazepine (P29), pipamperone with carbamazepine (P31), or pipamperone and lamotrigine (P38), led to stabilization. Anticonvulsants such as divalproex sodium, lamotrigine, or carbamazepine were associated with at least partial success, as was lithium in several cases (P25, P32, P36, P37, P45). Overall, antidepressants were poorly tolerated and ineffective.

### Catatonia

Sixteen of 56 cases reviewed (29%) were reported to have symptoms of catatonia, most commonly in the context of bipolar disorder (12 of 16, 75%). Several patients appeared to have acute triggers for their symptoms, including moving residences (P36, P37), or infection (P52, P56). Symptoms were highly variable but several patterns are noteworthy. Motor symptoms appeared to be common, with posturing and stereotypy, such as limb flexion, hunched posture, truncal instability, bradykinesia, upper extremity resting tremor, and stereotypic movements (*n* = 8). Some reports refer to “mild spastic paraparesis” (P2) or “intermittent spastic paraparesis of the upper left extremity” (P56) in patients with catatonia, which could be posturing or rigidity—characteristic motor signs of catatonia – and not true spasticity, particularly since spastic paraparesis would not describe signs in the upper extremities. Negativistic behaviors, stupor, and mutism were also thematic, with patients who stopped talking, moving, engaging in previously preferred activities, or refusing to eat, refusing to respond, and appearing apathetic (*n* = 7). Many patients were also described as exhibiting agitation (*n* = 6).

Regarding treatment of catatonia, benzodiazepines were used in some PMS cases with benefit (P30, P37, P56) but not in others (P50). Of note, electroconvulsive therapy (ECT) was typically effective when administered (P25, P32, P43). Antipsychotics were generally ineffective and poorly tolerated (P2, P25, P36), even inducing catatonia in at least one case (P36). It also appears that antidepressants and other serotonergic medications were associated with poor response and/or increased agitation in at least two cases (P32, P36). In many cases, lithium was used to treat the underlying bipolar disorder, often with success (P25, P31, P32, P36, P37, P50). Other anti-epileptic medications were commonly used, either in combination, or alone, and often with benefit. Among them, divalproex sodium appears to be the most commonly used and with the most consistent beneficial effects (P25, P31, P56).

### Psychosis

Seven of 56 patients (12.5%) were either diagnosed with schizophrenia (P16, P17), schizoaffective disorder (P15, P18), or unspecified psychosis (P43), or deemed to likely have a psychotic disorder upon our review (P6, P44). One of these cases (P6) first presented with psychosis (paranoid delusions and hallucinations) at 17 years old and at 38 years old was discovered to have neurofibromatosis type 2 due to ring chromosome 22. Symptoms in the cases were otherwise poorly described beyond using the term psychosis or providing the diagnosis without accompanying details. At least one case with psychosis (P43) had catatonia and responded to lorazepam after one episode and to ECT after another. Insufficient data was provided to otherwise review or draw any conclusions about treatment themes.

### Neurologic signs and progressive deterioration

Several individuals were reported with signs of what appears to be neurologic deterioration, such as development of parkinsonian signs, including resting tremor, bradykinesia, or mask facies, sometimes coupled with dysarthria, dysphagia, rigidity, or gait changes (P2, P3, P6, all with ring chromosome 22); unspecified tremor (P1, P21); gait changes (*n* = 12), including truncal or gait instability (P2, P3, P7, P52), ataxia (P34), paraparesis (P6, P20, P22, P27), or inability to walk (P12, P20, P22, P27, P28, P47); and swallowing difficulties (P14, P22). Some of the gait changes may be attributable to catatonia, which was mentioned in the original publication or considered to be a likely diagnosis on review (P2, P3, P7, P52), whereas in other cases they are likely a sign of a progressive neurological disorder (P6, P20, P22, P34), or related to an acute brain insult due to septic shock or status epilepticus (P27, P28, P47)*.* In one individual (P10), the cognitive and physical deterioration accompanied by seizures and sensorimotor polyneuropathy with onset at 12 years of age were secondary to juvenile onset metachromatic leukodystrophy.

## Discussion

In spite of the fact that fewer adolescent and adult patients with PMS are reported in the current literature compared to children, we identified 56 cases of PMS with neuropsychiatric decompensation, including 30 with loss of language, motor, or cognitive skills. While there are certainly ascertainment issues with this sample, these results suggest that neuropsychiatric decompensation and loss of skills in adolescence or adulthood could well be common in PMS and a part of the psychopathological phenotype of the disorder. It is important to note that neuropsychiatric decompensations occurred across a broad age range (9–51 years), but most commonly occurred between 16 and 20 years of age (Fig. 1). This observation is helpful to alert clinicians to this period of potentially increased risk, although it does not altogether allay concerns about later neuropsychiatric changes. The assessment and diagnosis of neuropsychiatric disorders in PMS is complicated by premorbid cognitive deficits, social communication impairment, and often restricted and repetitive behaviors. The Diagnostic and Statistical Manual for Mental Disorders, 5th edition [50] does not include modifications for patients with intellectual disability and limited language. Instead, the Diagnostic Manual – Intellectual Disability, Second Edition (DM-ID-2) [51] can be used for diagnosis and includes caregiver observations of behavior in addition to reducing the number of symptoms required for some diagnoses in order to remove criteria that require patients to describe their experiences.

### Loss of skills

Loss of skills can be defined in many ways and the word “regression” is interpreted to mean different things in different contexts. Typically, loss of skills is thought of as a prolonged loss of skills previously acquired and the term is consistently used in conjunction with a clear history of specific skills lost for a prolonged period. The amount of time defined as “prolonged” can vary, but typically a minimum of 3 months is required. Because skill loss can also occur in the context of neuropsychiatric disorders, it is critical to assess whether the loss is confined to the acute psychiatric episode or extends beyond when psychiatric symptoms return to baseline. Loss of skills and neuropsychiatric symptoms may also be more easily detected in higher functioning patients and therefore appears to be overrepresented among cases with smaller deletions or *SHANK3* mutations (see below). However, the extent of clinical information available in the literature to date makes it difficult to fully assess the nature of skill loss and whether losses would meet typical criteria for regression. Questions about the phenomenology of loss of skills and regression in childhood reported in PMS [4, 17–20] as compared to changes that occur in adolescence or adulthood remain. Finally, it is important to consider whether progressive increased severity of symptoms, with a decline in adaptive functioning, may implicate a neurodegenerative process or early onset of dementia.

Ten patients were reported with “atrophy” on brain imaging, most commonly involving the cerebral cortex, and in a few cases, subcortical structures (Table 2). These patients ranged in age from 19–70, and most were under age 45 when they had imaging. One was age 70, so cortical atrophy might be expected. Without serial scans showing a progressive change, it is hard to know if this is a meaningful change related to regression, and whether it is true atrophy or just a congenital small brain, perhaps due to PMS or other genetic changes in deletion carriers. If true progressive atrophy, this would raise the question of a secondary gene effect, particularly in deletion carriers, due to unmasking of a recessive variant in a gene in the deleted interval. Indeed, one of the individuals with diffuse cerebral and cerebellar atrophy at age 12 years had juvenile onset metachromatic leukodystrophy, also known as arylsulfatase A (ARSA) deficiency. It is important to note that white matter changes are not always obvious in adult and older juvenile cases of metachromatic leukodystrophy and these can present with psychiatric symptoms followed by gait changes such as spasticity or ataxia [52]. Thus, adolescents or adults with decompensation and 22q13.33 deletions including *ARSA* should be screened for this disorder (ARSA enzyme deficiency in blood leukocytes or urinary excretion of sulfatides, confirmed by biallelic pathogenic variants in *ARSA* on genetic testing).

**Table 2:** PMS patients with neuropsychiatric decompensation and atrophy on brain imaging

Case	Age at imaging	Imaging technique	Findings	Proposed diagnosis on review
2	27 y	CT	Diffuse mild cortical atrophy and hydrocephalus *ex vacuo*	Bipolar disorder with catatonia
10	12 y	MRI	Diffuse cerebral and cerebellar atrophy	Metachromatic leukodystrophy
11	52 y	CT	Cerebral atrophy, with multiple intracranial meningiomas	Bipolar disorder with catatonia, neurofibromatosis type 2
22	45 y	MRI	Normal, except for mild enlargement of the cisterna magna and central atrophy	Progressive neurological disorder
26	19, 25, and 41 y	CT	Corticosubcortical atrophy	Bipolar disorder
28	43 y	CT	Mild corticosubcortical atrophy	Bipolar disorder
31	70 y	MRI	Cortical atrophy, particularly in the frontal region, and subtle periventricular white matter changes	Bipolar disorder with catatonia
43	NA (between 20 and 46 y)	NA	Cerebral and cerebellar atrophy	Unspecified psychotic disorder with catatonia
45	42 y	MRI	Discrete loss of cerebral tissue predominantly in the left hemisphere with enlarged sulci	Bipolar disorder
56	NA (between 23 and 30 y)	MRI	Incipient cortical atrophy	Bipolar disorder with catatonia

*CT* computed tomography, *MRI* magnetic resonance imaging, *NA* not available, *y* years

### Bipolar disorder

According to the DSM-5, the diagnosis of bipolar disorder requires at least one lifetime manic episode defined as a distinct period of “persistently elevated, expansive, or irritable mood and persistently increased goal directed activity or energy, lasting at least 1 week and present most of the day, nearly every day” [50]. During this period, at least four symptoms are required, most of which may require some adaptation for persons with ID: (1) inflated self-esteem or grandiosity (may include exaggerated claims of accomplishment or skills for developmentally delayed people); (2) decreased need for sleep (or pronounced sleep disturbance); (3) more talkative than usual (or increased screaming, vocalizations, or other noise-making if minimally verbal); (4) flight of ideas or racing thoughts (when developmentally relevant); (5) distractibility (may manifest as diminished self-care skills in persons with ID or loss of productivity at work or day program); (6) increased goal-directed activity (people with ID may appear “sped up” or unable to sit still); (7) excessive involvement in pleasurable activities (in people with ID this may manifest as excessive masturbation, exposing self in public, or inappropriate sexual touching). If four or more distinct episodes of mania (or depression or hypomania) occur in the context of bipolar disorder during the past year, the course specifier of “rapid cycling” is applied [50].

Half the cases we reviewed met the criteria for bipolar disorder, including 12 with catatonia. Despite the challenges in reliably making the diagnosis in individuals with PMS who are intellectually disabled and often minimally verbal, the clinical themes that emerged were convincing. Irritability, mania, mood cycling, or mood dysregulation was commonly described, in addition to sleep disturbance, distractibility, and psychomotor hyperactivity. Many patients required hospitalization and loss of skills was commonly reported, most often in the language domain. Triggers were noted in some patients, including infection or menses; while insufficient evidence exists to establish any causal connections, the phenomenon may be useful for monitoring and possibly prevention in some cases. As is typical in PMS, treatment was challenging but antipsychotics were minimally effective and generally poorly tolerated. In some cases, the combination of a second generation antipsychotic (e.g., quetiapine, aripiprazole) with an anticonvulsant (e.g., divalproex sodium, carbamazepine, lamotrigine) was associated with good responses. Lithium should likewise be considered in cases of PMS with bipolar disorder. It would seem that in cases with an underlying mood cycling disorder, antidepressants are rarely associated with positive effects, and are often poorly tolerated. In all, these treatment strategies are generally aligned with guidelines for the management of bipolar disorder in the general population [53]. While our manuscript was under review, a case series was published documenting the longitudinal course and treatment of 24 individuals with PMS with accompanying neuropsychiatric symptoms [54]. Atypical bipolar disorder was diagnosed in 18 patients. In agreement with previous findings, treatment with a mood stabilizer (divalproex sodium or lithium), sometimes in conjunction with an atypical antipsychotic (olanzapine or quetiapine), was reported to result in gradual stabilization of mood and behavior in most individuals.

### Catatonia

The DSM-5 defines catatonia as a specifier diagnosed in the context of another medical condition or associated mental disorder (e.g., bipolar disorder). The clinical picture is characterized by at least three of the following symptoms: (1) stupor (i.e., no psychomotor activity; not actively relating to environment); (2) catalepsy (i.e., passive induction of a posture held against gravity); (3) wavy flexibility (i.e., slight, even resistance to positioning by examiner); (4) mutism (i.e., no, or very little, verbal response); (5) negativism (i.e., opposition or no response to instructions or external stimuli); (6) posturing (i.e., spontaneous and active maintenance of a posture against gravity); (7) mannerisms (i.e., odd, circumstantial caricature of normal actions); (8) stereotypy (i.e., repetitive, abnormally frequent, non-goal-directed movements); (9) agitation, not influenced by external stimuli; (10) grimacing; (11) echolalia (i.e., mimicking another’s speech); and (12) echopraxia (i.e., mimicking another’s movements) [50]. Of course, as the DM-ID2 notes, mutism, mannerisms, stereotypies, and grimacing can be features of ID, and echolalia can be a feature of ASD, so the history and time of onset of these symptoms is critical to delineate [51]. It is clear that catatonia often goes undiagnosed in individuals with intellectual and developmental disabilities [55] and yet appears to be a common feature of the neuropsychiatric presentation of PMS based on our review. The preponderance of females affected by catatonia was also notable (13 females versus 3 males), especially given the roughly equal sex ratio in PMS [56] and the fact that most youth diagnosed with catatonia are males [57, 58]. Thus, this observation needs to be confirmed in larger samples of individuals with PMS with a confirmed diagnosis of catatonia.

Benzodiazepines are typically the first line treatment for catatonia and were used in some PMS cases with benefit, albeit inconsistently. However, dosing information was not always available in the literature. Often response requires high doses (e.g., lorazepam 8 mg three times daily), with the caveat that dosing should always begin low (e.g., lorazepam 0.5–1 mg three times daily) and be titrated slowly with careful monitoring of vital signs. If benzodiazepines fail or provide only a partial response, ECT is considered the gold standard of care for catatonia [59] and was effective in most cases. Lithium should be considered in cases with bipolar disorder and catatonia, as response rates appeared relatively robust according to this review. Although commonly used, antipsychotics should be administered with caution in the patients given their limited benefit, pronounced side effects, and the potential risk of inducing catatonia. Despite this, some cases appeared to respond to the combination of second-generation antipsychotics (e.g., quetiapine) and anticonvulsants (e.g., divalproex sodium) or lithium. Antidepressants, especially in patients with mood cycling, show poor response and increased risk for symptom exacerbation.

### Psychosis

The diagnosis of schizophrenia requires that two or more symptoms during a significant proportion of at least one month (or less if successfully treated) be present to meet DSM-5 criteria, including (1) delusions, (2) hallucinations, (3) disorganized speech, (4) disorganized or catatonic behavior, and (5) negative symptoms. In addition, individuals must have at least one of the first three symptoms (delusions, hallucinations, disorganized speech). Level of functioning or self-care must be markedly below baseline functioning and there must be continuous signs of the disturbance for at least 6 months. If depressive or manic episodes occur concurrently, a diagnosis of schizoaffective disorder is more appropriate [50]. Although the DM-ID-2 does not delineate any significant adaptations for individuals with ID, criterion F of the DSM-5 does specify if there is a history of ASD or “a communication disorder of childhood-onset,” the diagnosis of schizophrenia requires the presence of delusions of hallucinations for at least 1 month (or less if successfully treated).

A minority of cases reviewed presented with psychotic symptoms and most reports provided too few details to reliably make the diagnosis of a primary psychotic disorder. Four cases were diagnosed explicitly with schizophrenia or schizoaffective disorder [28], all of whom had ID and were between the ages of 11 and 21 years-old. While it is likely that they experienced a psychiatric decompensation consistent with what is described in the other cases reviewed, confidence in the diagnosis of schizophrenia or schizoaffective disorder is undermined by the paucity of detail provided and the inherent challenges in making these diagnoses in intellectually disabled and developmentally delayed populations. No conclusions could be garnered regarding potential treatment of psychosis.

### Neurologic signs and progressive deterioration

Neurological signs observed in patients are diffuse and fall into categories of parkinsonism, tremor, gait changes due to ataxia, spasticity and others, and dysphagia as well as other descriptive changes. Some of these could be drug related (parkinsonian symptoms induced by antipsychotics, and tremor induced by lithium or divalproex sodium), related to neurological decompensation in a compromised brain with aging or illness, or a part of catatonia/psychiatric status. Others do appear to follow a persistent progressive neurodegenerative course (P20, P21, P22), which suggests a co-morbid neurological disorder. One patient (P10) is known to have such a disorder (metachromatic leukodystrophy) and others could have either this or another recessive disorder unmasked by the 22q13 deletion or a coincidental unrelated disorder. Onset of neurological conditions such as adult-onset metachromatic leukodystrophy in an individual with PMS could be particularly difficult to distinguish early in the disease course as later onset metachromatic leukodystrophy and other neurological diseases often present with psychiatric symptoms, and these symptoms may be difficult to interpret in a setting of ID and/or ASD.

### Role of *SHANK3*

Neurobehavioral decompensation, including bipolar disorder, catatonia, and loss of skills, was observed in cases with PMS regardless of the underlying genetic defect, consistent with a role of *SHANK3* in the psychopathological phenotype emerging as patients age. In fact, severe neuropsychiatric decompensation has been reported in 14 individuals with *SHANK3* point mutations [2, 4, 7, 28, 38–40]. These results indicate that *SHANK3* haploinsufficiency alone is sufficient to increase risk. These findings also suggest that patients with *SHANK3* mutations are overrepresented among individuals with PMS with neuropsychiatric decompensation or loss of skills. Whereas the proportion of patients with *SHANK3* variants in the PMS International Registry (which gathers genetic and clinical data from affected individuals around the world) is 8.6% (47 out of 546 with a genetically confirmed diagnosis), it rises to 25% (14 of 56) among the cases reviewed here (Fisher’s exact test, *p* = 0.00057). This could be related to the fact that some individuals with *SHANK3* mutations or small deletions develop phrase speech and can have less severe cognitive and motor deficits compared to individuals with large 22q13.3 deletions, making it easier to recognize the psychiatric disorders and loss of skills. Alternatively, the higher level of functioning could render them more vulnerable to environmental and medical stressors. The mechanisms through which reduced expression of SHANK3 is associated with neuropsychiatric decompensation and loss of skills are unclear.

### Predisposing and precipitating factors

In several patients, extensive neurologic and metabolic investigations were non-diagnostic. In the majority of cases, no apparent cause could be identified; in others, the symptoms appeared after acute infections (P22, P52, P39, P52, P56), or presumably stressful environmental changes, such as being transferred to a new residential institution in five individuals (P13, P14, P33, P36, P37), or an institutional reorganization in another (P45). In three cases, the neurologic deterioration appears to have been related either to an increase in seizures, despite treatment (P20), or following a severe status epilepticus (P28, P47). In one individual (P10), the cognitive and physical deterioration appears to be secondary to metachromatic leukodystrophy [25], an autosomal recessive disorder characterized by progressive demyelination of peripheral and central nervous systems and caused by mutations in the arylsulfatase A (*ARSA*) gene on chromosome 22q13.33. Patients with deletions extending proximal to *SHANK3* have one missing copy of *ARSA* and may develop metachromatic leukodystrophy in the presence of a pathogenic mutation in the remaining *ARSA* allele. However, the loss of both copies of the *ARSA* gene would be a rare event, expected in about 1/100–1/200 patients with PMS and a deletion involving *ARSA* (based on the estimated carrier frequency of *ARSA* mutations) [52]. Despite this expected frequency, there are only a handful of cases reported in the literature, and we know of no diagnosed cases in the PMS Foundation or national PMS associations. Therefore, metachromatic leukodystrophy is not expected to be a significant etiological factor in most patients with PMS exhibiting a regression phenotype, although the possibility that this disorder may be currently underdiagnosed cannot be excluded. Another slowly progressive autosomal recessive neurological disorder affecting white matter and causing progressive gait, fine motor, and cognitive disturbance, megalencephalic leukoencephalopathy with subcortical cysts due to biallelic *MLC1* mutations, can also be unmasked by 22q13.33 deletions. This has been seen in one instance (unpublished patient of EBK); however, none of the neuroimaging described here was consistent with that disorder.

Five patients in this series (P3, P6, P11, P32, and P51), all with a ring chromosome 22, developed neurofibromatosis type 2 associated tumors, diagnosed in adolescence or adulthood. Ring chromosomes are unstable during somatic mitoses and are prone to secondary rearrangements and subsequent loss. As a result, individuals with ring chromosome 22 often exhibit mosaic monosomy 22. In the cells that lost the ring chromosome, a somatic mutation in the remaining *NF2* gene results in tumor development; this is referred to as the two-hit model [60]. However, these tumors are not expected to be the cause of regression or neuropsychiatric decompensation in the majority of cases, since individuals with neurofibromatosis type 2 not associated with ring chromosome 22 and loss of *SHANK3* do not exhibit an increased rate of psychopathology [61].

Anecdotal reports from families often describe acute events as frequent triggers, and when addressed, may lead to rapid resolution. As such, gastrointestinal disturbances (e.g., gastroesophageal reflux and constipation), urinary tract infections or retention, dental caries, ear infections, ovarian cysts, and uterine fibroids or tumors, should always be ruled out. Hormonal changes during the menstrual cycle may also contribute to psychiatric symptomatology and can sometimes be addressed by regulating menses using contraceptive medication.

### Similar clinical presentations in other neurodevelopmental disorders

As older patients with genetic disorders are being diagnosed and assessed, we are gleaning insights into phenotypes throughout the lifespan. In both PMS and in other genetic disorders, neuropsychiatric deterioration appears to be more frequent than previously thought. In particular, regression, bipolar disorder, psychosis, and catatonia have been described in several other neurodevelopmental disorders associated with specific genetic defects. Kleefstra syndrome is caused by deletions or mutations of the *EHMT1* gene, encoding a histone methyltransferase, and, like PMS, presents with ID, ASD, severe speech deficits, and hypotonia, in addition to distinctive facial features. At least six individuals with Kleefstra syndrome have been reported with severe behavioral regression developing during adolescence or adulthood, with periods of apathy and catatonia-like behaviors [62–64]. Individuals with Kleefstra syndrome also exhibit a high prevalence of depression, psychosis, and obsessive–compulsive disorder, with a general decline in functioning in all patients older than 18 years, usually preceded by severe sleep problems [65]. This regression has been hypothesized to be due to an often unrecognized psychotic episode, not treated adequately [65, 66], but certainly all these late onset symptoms could be the course of the disease and represent developmental changes in symptom susceptibility. 22q11.2 deletion syndrome (also known as velocardiofacial or DiGeorge syndrome) is also frequently associated with psychotic disorders, including a 25-fold increased risk of developing schizophrenia [67], typically emerging in late adolescence/early adulthood. The onset of psychosis is commonly preceded by cognitive decline [68]. Catatonia may be a relatively common finding in individuals with 22q11.2 deletion syndrome but often goes unrecognized [69]. In contrast, the prevalence of bipolar disorder does not appear to be increased compared to the general population [67].

Behavioral regression, bipolar disorder, psychosis, and catatonia have also been reported in patients with *MBD5* haploinsufficiency (also known as autosomal dominant mental retardation 1 or 2q23.1 deletion syndrome) [70, 71]; psychosis and catatonia are known to occur in a fraction of patients with Down syndrome [72–75]; and several instances of regression, psychosis/schizophrenia, and bipolar disorder were described in Tatton-Brown-Rahman syndrome, an overgrowth ID syndrome caused by *DNMT3A* variants [76]. High rates of catatonia have also been reported in individuals with idiopathic autism [77, 78] as well as in those with ID [79], suggesting shared pathophysiological mechanisms. Further research is needed to study the prevalence of neuropsychiatric disorders across the lifespan in individuals with neurodevelopmental disorders of different etiologies and determine in which of these disorders neuropsychiatric disorders emerge more frequently than in the general population indicating an enhanced susceptibility. Possibly disorders with proven enhanced susceptibility will have overlapping molecular mechanisms that could provide clues to the underlying neuronal pathways promoting this susceptibility.

## Limitations

The results from this review must be interpreted with caution due to several limitations. First, the cases reviewed may not be representative of the PMS population in its entirety. Due to ascertainment bias and underdiagnosis, it is impossible to estimate the overall prevalence of neuropsychiatric decompensation or loss of skills in PMS. Second, while clearly dramatic neuropsychiatric changes and loss of skills occur, the precise nature and extent of symptoms remain challenging to elucidate because many reports have limited descriptions of the subjects. While other reports present a more complete clinical evaluation, descriptions are mainly retrospective in nature. In particular, as noted, details about loss of skills and “regression” in most of the case reports do not clarify baseline levels of acquired skills or time course after skill loss. Likewise, psychotic symptoms were mentioned often in reports but too few details were available to reliably make the diagnosis of a primary psychotic disorder in most cases. In addition, it is challenging to establish a diagnosis in many cases based on the paucity of details provided in some of the original case reports and the review nature of our study design. Finally, regarding treatment, the number of patients receiving a given treatment was very limited and different doses and durations of treatment were applied. Treatment responses were also not assessed using standardized or validated measures. As such, insufficient data were available to draw firm conclusions about treatment themes. However, ongoing work is dedicated to establishing formal consensus treatment guidelines based on available evidence from the literature and expert clinician experience.

## Conclusions

In conclusion, the need for more systematic follow-up of the patients with PMS is crucial to facilitate our knowledge of disease progression but also, and more importantly, to optimize patient management. Indeed, it is evident that clinicians and caretakers need to be vigilant for loss of skills and neuropsychiatric changes in adolescents and adults with PMS, including the development of bipolar disorder and catatonia. The possibility of progressive neurological disorders needs to be considered, particularly in patients with 22q13 deletions that may unmask a recessive mutation. As successful interventions are identified, these approaches should become a part of the management of PMS. Until such time that formal consensus treatment guidelines are established, results from this review suggest that antidepressants and antipsychotic medications should be used with caution in PMS. And since loss of *SHANK3* alone is sufficient to lead to susceptibility to loss of skills and neuropsychiatric decompensation, model systems should be studied over the lifespan and in the context of additional stressors to begin to dissect the pathobiology of regression in PMS and help in the development of novel interventions.

In an attempt to address some of the current treatment challenges highlighted in this review, the PMS Neuropsychiatric Consultation Group (PMS-NCG) was formed and aims to provide multidisciplinary consultation to geographically dispersed physicians, to support them in providing the best possible care to patients with PMS. This initiative utilizes an established model for knowledge dissemination called ECHO (https://echo.unm.edu/), which is based on video-conferencing case consultation with teams of experts and local providers meeting regularly to discuss case management. Information about clinical outcomes is also collected after ECHO consultations to inform future treatment guidelines. For more information, providers can visit the PMS Foundation website (https://www.pmsf.org/echo-project/).

## Data Availability

Not applicable

## Abbreviations

ASD: Autism spectrum disorder
ECT: Electroconvulsive therapy
FISH: Fluorescence in situ hybridization
ID: Intellectual disability
IQ: Intellectual quotient
PMS: Phelan-McDermid syndrome
